# Oxidized phospholipids in the macula increase with age and in eyes with age-related macular degeneration

**Published:** 2007-05-23

**Authors:** Mihoko Suzuki, Motohiro Kamei, Hiroyuki Itabe, Kazuhito Yoneda, Hajime Bando, Noriaki Kume, Yasuo Tano

**Affiliations:** 1 Department of Ophthalmology, Osaka University Medical School, Suita, Japan; 2 Department of Biological Chemistry, School of Pharmaceutical Science, Showa University, Tokyo, Japan; 3 Department of Ophthalmology, Kyoto Prefectural University of Medicine, Kyoto, Japan; 4 Ophthalmic Research, Cole Eye Institute, The Cleveland Clinic Foundation, Cleveland, OH; 5 Department of Cardiovascular Medicine, Graduate School of Medicine, Kyoto University, Kyoto, Japan

## Abstract

**Purpose:**

There is good evidence that oxidative stress is involved in the pathogenesis of age-related macular degeneration (AMD). Because AMD has risk factors and histopathology similar to with atherosclerosis, we hypothesized that oxidized phospholipids, which contribute to the pathogenesis of atherosclerosis, would accumulate in the eyes of AMD patients. To test this hypothesis, we investigated whether oxidized phospholipids were present in normal eyes and whether the level changed with increasing age. We then, we determined whether the levels of oxidized phospholipids were higher in eyes with AMD.

**Methods:**

Twenty normal human donor eyes and six eyes with AMD were studied. Immunohistochemistry was performed on a tissue strip from the macular region using an antibody against oxidized phosphatidylcholine. Western blot analysis was also performed on proteins extracted from the posterior retina of donor eyes. The immunoreactivity of the specimens and the bands were quantified with NIH image software.

**Results:**

Immunohistochemistry showed oxidized phosphatidylcholine was present in the photoreceptors and retinal pigment epithelium of the normal human macular area, and their levels increased with age. Eyes with AMD showed more intense immunoreactivity for oxidized phospholipids than age-matched normal eyes.

**Conclusions:**

These findings suggest that oxidative stress is involved in the pathogenesis of AMD possibly by oxidizing phospholipids in the photoreceptors as demonstrated in the arterial intima of patients with atherosclerosis. It is likely that controlling oxidation of phospholipids may be a potential treatment for AMD.

## Introduction

Age-related macular degeneration (AMD) is a leading cause of blindness in the elderly population of industrialized countries [1]. Although several therapies including photodynamic therapy [2] and anti-VEGF therapy [3] have been recently developed, they can only stabilize the already reduced vision. An incomplete understanding of the pathogenesis of AMD may be one reason for the limited success of the therapies currently used [4,5].

AMD is characterized by a progressive degeneration of the neurosensory retina, retinal pigment epithelium (RPE), and choriocapillaris in the macular area. Because the macula, which is subjected to the highest levels of cumulative irradiation, has the highest level of oxygen consumption and has a unique composition of fatty acids [6], oxidative stress has been suggested to be part of the initial pathogenic mechanism of AMD [7-9].

In vitro studies have shown that feeding oxidized photoreceptor outer segments to cultured RPE cells increased the number of lipofuscin granules in the RPE cells [10]. Excess accumulation of lipofuscin and lysosomes in RPE cells can lead to drusen formation, an initial clinical change in AMD [11]. However, a direct relationship between the oxidative changes and the development of AMD has not been reported.

In a histopathological study, Curcio and co-workers demonstrated an age-related accumulation of cholesterol esters in Bruch's membrane similar to that observed in the arterial intima [12]. Killingsworth et al. also observed that macrophages and phospholipid-containing debris were co-localized in Bruch's membrane in eyes with AMD [13]. Because these pathological changes in AMD are similar to those seen in atherosclerosis [12-17], and because atherosclerotic changes may contribute to the pathogenesis of AMD [18,19], we hypothesized that oxidized phospholipids would accumulate in the eyes of patients with AMD as has been demonstrated in the arteries of patients with atherosclerosis [20]. The presence of oxidized phospholipids in the macula region has not been reported, so we first investigated whether they were present in normal eyes and whether the level changed with increasing age. Then, we determined whether oxidized phospholipids were increased in eyes with AMD to test our hypothesis.

## Methods

### Reagents

All chemicals, unless otherwise stated, were purchased from Vector Laboratories (Burlingame, CA). A monoclonal IgM antibody against oxidized low-density lipoprotein (LDL), FOH1a/DLH3, that specifically recognizes oxidized phosphatidylcholine (Ox-PC) was generated in one of the coauthor's laboratory by immunizing a mouse against homogenates of human atheroma [21].

### Donor tissues

Twenty normal human donor eyes and six eyes with AMD were used. The normal eyes were obtained from the Cleveland Eye Bank (Cleveland, OH) or the National Disease Research Interchange (Philadelphia, PA). The AMD donor eyes were obtained from patients clinically diagnosed with AMD, and the donors were registered through the Eye Donor Program of The Foundation Fighting Blindness (Hunt Valley, MD). Eyes were frozen in liquid nitrogen after enucleation.

### Tissue preparation

A 10x12 mm rectangular section was cut from the posterior pole or the periphery of each frozen or fixed globe according to a described method described by Itabe, et al. [5].

A 2 mm-wide strip of the retina-RPE-choroid-sclera complex, centered on the fovea, was dissected from the section. It was embedded in optimum cutting temperature (OCT) compound (Sakura Finetechnical Co, Ltd, Tokyo, Japan), and 8 μm thick cryosections were cut for immunohistochemistry. The remaining tissue was used for western blot analysis and other experiments.

### Immunohistochemistry

Indirect immunohistochemistry was performed on cryosections of the human tissue using the avidin-biotin complex immunoperoxidase technique. Briefly, after fixing the sections in cold 4% formaldehyde, the endogenous peroxidase activity was blocked by NaIO_4_. The specimens were incubated with 5% bovine serum albumin (BSA) in phosphate buffered saline (PBS) to block nonspecific immunoreaction and then exposed to a monoclonal antibody against oxidized phospholipids, DLH3 [21], at a dilution of 1:100 followed by incubation with a biotinylated horse anti-mouse IgM antibody. The sections were then incubated with streptoavidin-biotin complex labeled with peroxidase. The immunoreactivity was made visible by 3-amino-9-ethylcarbazole (AEC, Vector Laboratories). Sections incubated with nonimmune mouse IgM as a primary antibody served as negative controls. Three serial sections of each eye were analyzed, and the examiners were masked to the age of the eyes.

For quantitative immunohistochemical analysis, the density of the staining in the photoreceptors and the immunostained area in the RPE was measured with the NIH image software based on the threshold technique. The correlations between the age and the density of staining were determined with the Spearman rank correlation coefficient. Differences between AMD and age-matched normal tissue were analyzed with the Mann-Whitney test.

### Western blot analysis

To examine which molecules were recognized by DLH3, we performed western blot analysis with the retinal lysates from normal donor eyes. Proteins were obtained for the western blots from a 10x10-mm wide square of retina from each donor eye. Tissues were lysed in a 10 fold volume of extraction buffer (200 mM NaCl, 1% Triton X-100, 500 mM Tris-Hcl, pH 7.6) using a sonicator (four bursts of 5 s). After centrifugation at 15 000 rpm for 15 min, the supernatant was collected. The protein concentration of each sample was measured using bicinchoninic acid (BCA; Pierce, Rockford, IL) and BSA as a reference standard.

Then 25 μg of total protein was carefully measured out, boiled in SDS sample buffer (62.5 mM Tris-HCl, pH 6.8, 25% glycerol, 0.01% bromophenol blue, and 2% SDS), separated by SDS-PAGE, and transferred to Immobilon-P membranes (Millipore, Bedford, MA) using a Bio-Rad Semi-Dry Electrophoretic Transfer Cell (20 min transfer at 18 volts). After the transfer, the membranes were blocked with 2% BSA in PBS for 1 h at room temperature, and the gels were stained with Gelcode (Pierce) to determine the isolated proteins.

The membranes were then incubated with the primary antibody for Ox-PC, DLH3, at 1:1000 dilution overnight at 4 °C. The membranes were washed with 0.05% Tween in PBS and then incubated with Alexa Fluor® 568 goat anti-mouse IgM in 0.05% PBS/Tween for 1 h at room temperature. The western blot was imaged with Typhoon 8600 (GE Healthcare Bio-Sciences Corp., Piscataway, NJ) using a 532 nm excitation filter. The images were displayed using the Image Quant^TM^ software (GE Healthcare Bio-Sciences Corp). The densities of the bands were quantified with the NIH image software. The correlations between the age and densities were determined with the Spearman rank correlation coefficient.

## Results

### Immunohistochemistry

**Figure 1:**
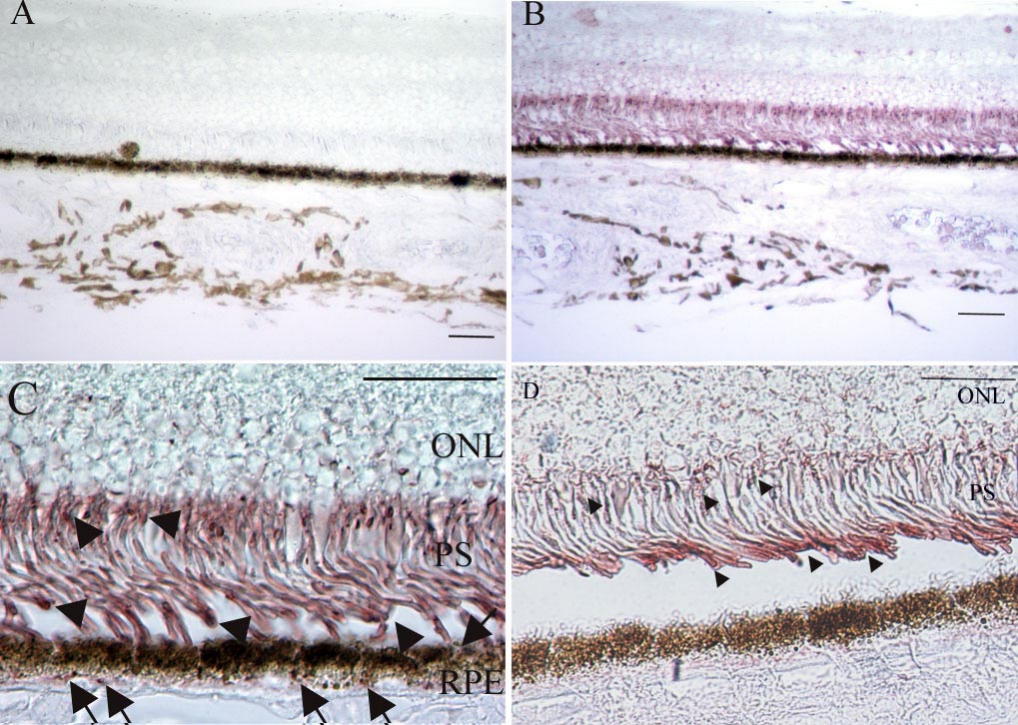
Immunohistochemical examination for oxidized phospholipids of a normal eye. **A**: Retinal section from the macula area of a normal eye. Negative control with non-immunized mouse IgM as a primary antibody. Original magnification, x100. Scale bar represents 50 μm. **B**: Retinal section from the macula area of a normal eye. Oxidized phospholipids are detected in the inner and outer retina and choroid. Original magnification, x100. Scale bar represents 50 μm. **C**: Retinal section from the macula area of a normal eye. Oxidized phospholipids are seen mostly in the photoreceptors (PS) and retinal pigment epithelium (RPE). Immunohistochemical analysis of the photoreceptor inner and outer segments demonstrated diffuse immunoreactivity (arrowheads). Both the basal and apical sides of the RPE cells are primarily stained (arrows). Original magnification, x600. Scale bar represents 50 μm. **D**: Retinal section from the peripheral area of a normal eye. Oxidized phospholipids are seen in the photoreceptor inner and outer segments (arrows). Immunoreactivity was less intense than in the macular area. Original magnification, x600. Scale bar represents 50 μm. ONL indicates outer nuclear layer

Positive staining for oxidized phospholipids was observed in the photoreceptor inner and outer segments and RPE cells in normal human donor eyes (Figure 1). Immunoreactivity in the photoreceptor inner and outer segments in the macular area was higher than in the peripheral area. Both the basal and the apical sides of the RPE cells were primarily stained. There was no difference between fixed and frozen globes in the stain pattern with this antibody.

**Figure 2:**
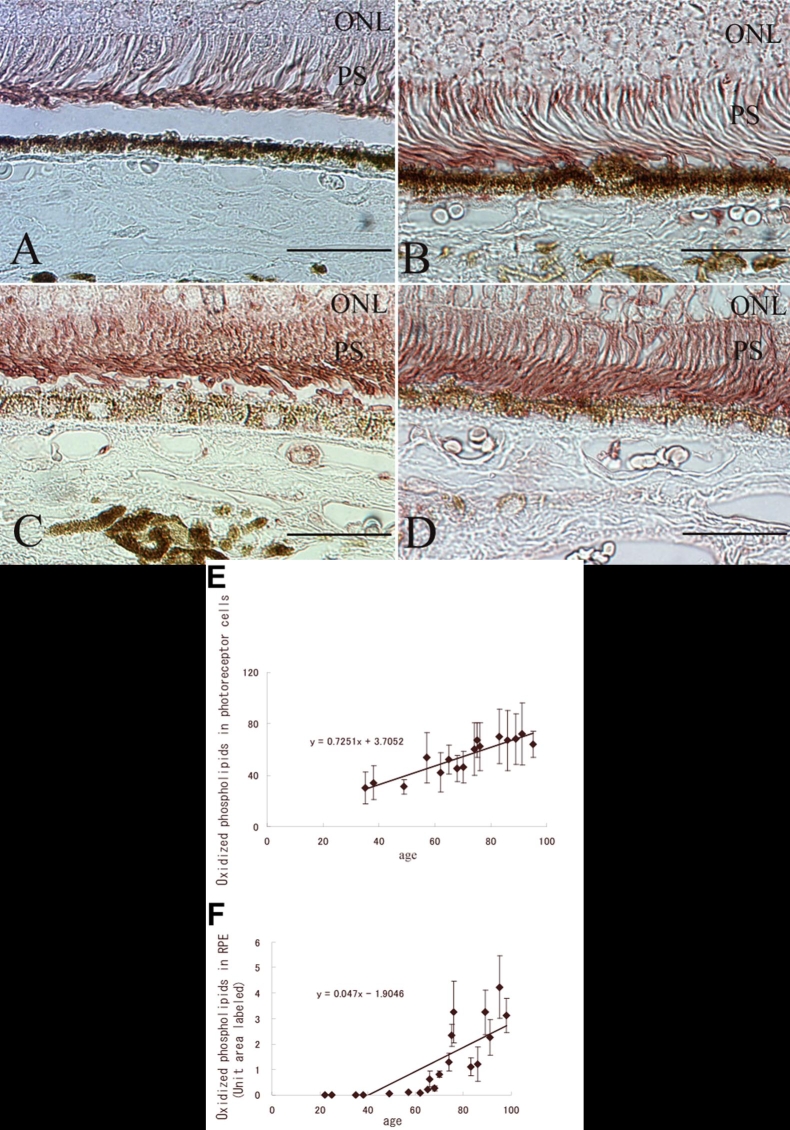
Increased oxidized phospholipids immunostaining with advancing age in normal eyes. Sections were from the following: (**A**) 38-year-old donor; (**B**) 57-year-old donor; (**C**) 65-year-old donor; and (**D**) 76-year-old donor. Original magnification, x600. Scale bar represents 50 μm. The eyes from the younger donors, especially the 38-year-old donor, is immunostained much less than the older eyes. **E**: Threshold values of immunoreactivity for oxidized phospholipids in the photoreceptors. **F**: Percentage area of field immunostained for oxidized phospholipids in the retinal pigment epithelium (RPE). ONL indicates outer nuclear layer, and PS represents photoreceptors.

A comparison of the immunoreactivity of eyes of different ages showed that eyes from younger donors (under 60 years) showed less intense immunoreactivity than eyes from older donors (60 years or older) in both the photoreceptors and RPE cells (Figure 2A-D). The immunological staining density was significantly correlated with age in the photoreceptors (r=0.8911; n=16; p<0.01; Spearman rank correlation coefficient; Figure 2E) and RPE (r=0.9283; n=20; p<0.01; Spearman rank correlation coefficient; Figure 2F). Oxidized phospholipids in the photoreceptors were analyzed in only 16 normal eyes, because the photoreceptors of the remaining four eyes were not well-preserved.

**Figure 3:**
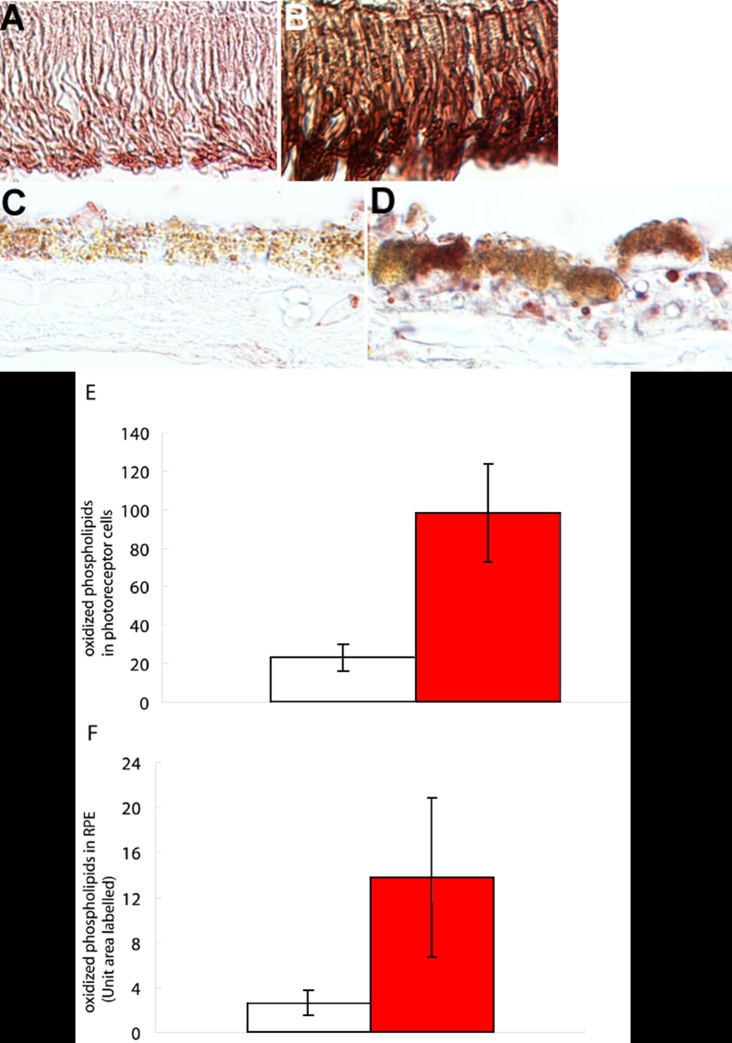
Oxidized lipoprotein immunostaining is more intense in eyes with age-related macular degeneration than in age-matched normal eyes. **A**: Photoreceptors (PS) of 75-year-old normal donor. **B**: PS of 70-year-old age related macular degeneration (AMD) donor, **C**: Retinal pigment epithelium (RPE) of the same normal donor as **A**. **D**: RPE of the same AMD donor as **B**. Eyes with AMD show more intense immunoreactivity for oxidized phospholipids than age-matched normal eyes in both the photoreceptors and RPE cells. In the AMD eyes, note that the areas with continuous soft drusen show immunoreactivity (arrowheads). Original magnification, x600. Scale bar represents 50 μm. **E**: Mean threshold values of immunoreactivity for oxidized phospholipids in the photoreceptors of non-AMD age-matched control donor eyes (clear column) and AMD (filled column). **F**: Mean percentage area of field immunostained for oxidized phospholipids in the RPE of non-AMD age-matched control donor eyes (clear column) and AMD (filled column).

Eyes with AMD showed more intense immunoreactivity than that in age-matched normal eyes. Immunostaining was more prominent in the RPE cells than in the photoreceptors (Figure 3). The soft drusen in the AMD eyes were also immunopositive for oxidized phospholipids, which suggested that the oxidized compounds may be more difficult to digest by the lysosomes in the RPE cells and were exocytosed to Bruch's membrane. The staining intensity in the photoreceptors was higher in AMD eyes than in age-matched normal eyes (p<0.01; n=6; Mann-Whitney test; Figure 3E). The immunostained areas were stained more strongly in AMD eyes than in age-matched normal eyes (p<0.01; n=6; Mann-Whitney test; Figure 3F).

### Western blot analysis

**Figure 4:**
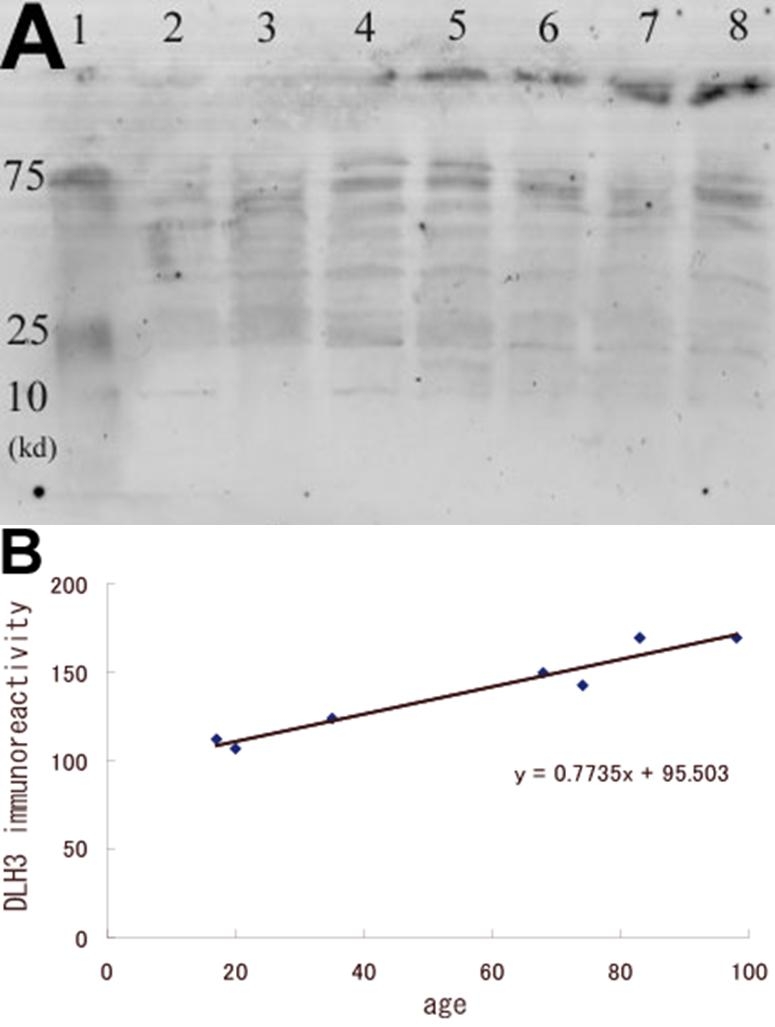
Comparison of the level of the oxidized phospholipids using Western blot analysis with 25 μg of proteins of the samples from normal donor eyes of different ages. **A**: Several bands that probably represent phospholipids containing oxidized phosphatidylcholines can be seen. The intensity of the bands increases with age. Lane 1 is the: molecular weight marker, lanes 2-8: samples from donors aged 17, 20, 35, 68, 74, 83, and 98 years old, respectively. **B**: Densitometric measurements of the FOH1a/DLH3 immunoreactivity. A significant correlation can be seen between age and the density of staining (r=0.89).

Western blot analysis showed several bands that might represent molecules containing oxidized phosphatidylcholines just as atherosclerosis lipoproteins show several bands or smear-staining [22]. The density of the bands increased with age (r=0.8928; n=7; p<0.01; Spearman rank correlation coefficient; Figure 4B). The phospholipids that formed the plasma membrane exist partly as lipoproteins, a complex of phospholipids and proteins. Phospholipids, including phosphatidylcholine, can immunoreact with FOH1a/DLH3 when the phosphatidylcholines are oxidized, because DLH3 recognizes oxidized phosphatidylcholines.

## Discussion

Although strong evidence has accumulated that oxidative stress plays a key role in the pathogenesis of AMD [4,7-9], it has not been directly demonstrated how the oxidative stress contributes to the development and progression of the disease. For example, it has not been established whether oxidized materials are present in the appropriate areas of eyes with AMD. Our results confirmed that oxidized phospholipids were indeed present in the photoreceptors and RPE cells in the macula region of the normal eyes, and the quantity increased with age. In addition, eyes with AMD showed more intense immunoreactivity to oxidized phospholipids than age-matched normal eyes. These results support our hypothesis that oxidized phospholipids accumulate in the eyes of patients with AMD. These findings would then indicate that oxidative damage of the photoreceptors and RPE cells may be the mechanism for the changes induced in eyes with AMD. We suggest that the oxidation of phospholipids in the photoreceptors may lead to incomplete digestion of the photoreceptor discs phagocytosed by the RPE. This may result in the accumulation of lipofuscin in the RPE and subsequently drusen formation. Finally, the accumulation of lipofuscin could lead to RPE dysfunction, which would then cause the death of photoreceptor cells.

The degree of positive immunoreactivity in the photoreceptor inner and outer segments in the macular area was higher than in the peripheral area. The photoreceptors are probably subject to oxidative modifications because of their unique structure. The outer segments consist of continuous infoldings of the plasma membrane, which means that the photoreceptors contain much more phospholipids, which include high polyunsaturated fatty acids that are subject to oxidative modifications, than other cells of the retina [6]. Additionally in the macular area, the level of oxygen from the choroidal circulation could be higher [23] because photoreceptor density is higher than the periphery [24]. The results of a study also showed that the level of oxidized LDL in the plasma is increased in patients with AMD possibly in relation to paraoxonase gene polymorphisms [25].

Considering that AMD is a disease affecting the photoreceptors, RPE cells, and choriocapillaris, it is interesting that oxidized phospholipids were detected almost exclusively in the photoreceptors and RPE cells and were not observed in the inner layers of the retina. RPE cells continuously ingest shed photoreceptor discs at their apical border, form phagosomes within the cytoplasm, breakdown the fragments of the outer segment discs in their lysosomes, and exocytose the degraded materials on the basal side. Because an accumulation of oxidized compounds inhibits the function of phagosomes in the RPE [26,27], incompletely digested photoreceptors containing oxidized phospholipids are probably exocytosed and observed as debris. This is consistent with our results that oxidized phospholipids were found in the RPE cells and in soft drusen. Our findings confirm and expand the results of previous studies reporting that lipoproteins and phospholipids are contained in drusen [28,29], and that oxidation of these materials is strongly related to AMD [7,8].

We are not aware of any published study that has demonstrated the presence of oxidized phospholipids in the macula, probably because an antibody to detect oxidized phospholipids has not been commercially available. The antibody we employed recognizes oxidized phosphatidylcholines [30], and has been used to detect oxidized LDL by immunohistochemistry and ELISA [31,32]. The phosphatidylcholines are one of the most abundant phospholipids in the photoreceptors and account for approximately one-third of the total lipids in the photoreceptors [25]. We tried sandwich ELISA with DLH3 and anti-apoB antibodies to quantify the oxidized lipoproteins in the retina, but we could not obtain reliable results probably because apoB does not exist in the photoreceptors. Establishing a quantitative method to evaluate the amount of oxidized phospholipids in the retina remains as a challenge for the future.

In the early stage of atherosclerosis, endocytosis of oxidized LDL including oxidized phospholipids by macrophages and subsequent foam cell transformation are key events [33]. Because macrophages also accumulate in the AMD lesions [14,34], and the presence of oxidized phospholipids in AMD lesions have been shown in this study, macrophages may take up oxidized phospholipids as observed in atherosclerosis. We are investigating whether macrophages in AMD lesions express scavenger receptors for oxidized phospholipids.

In conclusion, our results demonstrated that oxidized phospholipids were present in the normal human macular region, and their levels increase with age as in the arterial intima in atherosclerosis. In addition, eyes with AMD have more intense immunoreactivity for oxidized phospholipids than age-matched normal eyes. These findings indicate that oxidative stress is probably involved in the pathogenesis of AMD. Our findings are consistent with the suggestion that supplementation with antioxidants, vitamins, and minerals may reduce the risk of developing AMD [35].
